# Association Between Complete Right Bundle Branch Block and Vascular Endothelial Function

**DOI:** 10.1016/j.jacasi.2026.02.016

**Published:** 2026-04-11

**Authors:** Aya Mizobuchi, Tatsuya Maruhashi, Yusuke Saito, Takayuki Yamaji, Takahiro Harada, Farina Mohamad Yusoff, Shinji Kishimoto, Masato Kajikawa, Ayumu Nakashima, Yukihito Higashi

**Affiliations:** a Department of Regeneration and Medicine, Division of Radiation Medical Science Research Institute for Radiation Biology and Medicine, Hiroshima University, Hiroshima, Japan; b Center for Radiation Disaster Medical Science, Research Institute for Radiation Biology and Medicine, Hiroshima University, Hiroshima, Japan; c Division of Regeneration and Medicine, Medical Center for Translational and Clinical Research, Hiroshima University Hospital, Hiroshima, Japan; d Department of Nephrology, Graduate School of Medicine, University of Yamanashi, Yamanashi, Japan

**Keywords:** complete right bundle branch block, flow-mediated vasodilation, nitroglycerine-induced vasodilation, vascular function

## Abstract

**Background:**

Complete right bundle branch block (CRBBB) is a common electrocardiographic finding even in healthy people and generally has a good prognosis. However, recent studies have shown that CRBBB is related to arteriosclerosis and cardiovascular risk.

**Objectives:**

This study aimed to assess the association between CRBBB and vascular function.

**Methods:**

This study included a total of 2,313 individuals. After applying the exclusion criteria, 1,308 individuals were included in the final analysis. We evaluated the endothelial function assessed by flow-mediated vasodilation and vascular smooth muscle function assessed by nitroglycerine-induced vasodilation in 55 individuals with CRBBB (34 men; mean age: 62.6 ± 16.6 years) and 1,253 individuals with normal QRS duration (527 men; mean age: 66.6 ± 14.0 years).

**Results:**

Flow-mediated vasodilation was significantly lower in patients with CRBBB than that in those with normal QRS duration (3.2% [1.6%-4.7%] vs 4.6% [2.6%-6.7%]; *P* < 0.001). In the multivariable analysis, CRBBB was significantly associated with endothelial dysfunction (OR: 1.84; 95% CI: 1.01-3.36; *P* = 0.047). Although nitroglycerine-induced vasodilation was significantly lower in individuals with CRBBB than in those with normal QRS duration (11.5% [7.9%-14.9%] vs 13.1% [9.6%-17.3%] *P* = 0.024), no significant association was observed between CRBBB and vascular smooth muscle dysfunction based on the multivariable analysis (OR: 1.09; 95% CI: 0.53-2.22; *P* = 0.816).

**Conclusions:**

CRBBB was shown to be associated with endothelial dysfunction. These findings suggest that CRBBB should not be regarded as a benign electrocardiography finding from a vascular function standpoint (A study of clinical usefulness of vascular function; UMIN000039512)

Complete right bundle branch block (CRBBB) is an electrocardiographic finding that results from an interruption in the right bundle branch of the cardiac conduction system. Although CRBBB is more common in men and older adults, it can also be observed in healthy young adults. CRBBB has long been considered an electrocardiographic finding with no clinical significance or cardiovascular risk.^1^, ^2^, ^3^ However, recent large-scale cohort studies have reported associations between CRBBB and various comorbidities, and adverse prognostic implications.^4^, ^5^, ^6^, ^7^, ^8^ Despite these findings, conclusive evidence for a statistically significant association between asymptomatic CRBBB and cardiovascular events remains elusive. Accordingly, major clinical guidelines have not provided specific recommendations regarding cardiovascular risk assessment or management strategies in patients with asymptomatic CRBBB without significant QRS prolongation.^9^, ^10^, ^11^, ^12^

Flow-mediated vasodilation (FMD) is a marker of endothelial function. It indirectly assesses nitric oxide production from the endothelium by measuring changes in arterial diameter after reactive hyperemia. Moreover, endothelial dysfunction plays a crucial role in the development, maintenance, and progression of cardiovascular disease.^13^, ^14^, ^15^, ^16^ Several lines of evidence indicate that endothelial function, as assessed by FMD, serves as a predictor of future cardiovascular events.^13^^,^^17^, ^18^, ^19^

Nitroglycerine-induced vasodilation (NID), which is considered a marker of vascular smooth muscle function, assesses the vasodilatory response after the sublingual administration of nitroglycerine. Our previous study has shown that NID also serves as an independent predictor of future cardiovascular events.^18^

Given the prognostic value of vascular function tests for cardiovascular events, investigating the associations of CRBBB with FMD and NID may provide further insights into the potential association between CRBBB and cardiovascular risk.

However, to date, no studies have yet investigated the relationship between CRBBB and vascular function. Thus, this study aimed to investigate the association of CRBBB with endothelial function assessed by FMD and vascular smooth muscle function assessed by NID in a large cohort of well-characterized individuals.

## Methods

### Study participants

**Figure 1:**
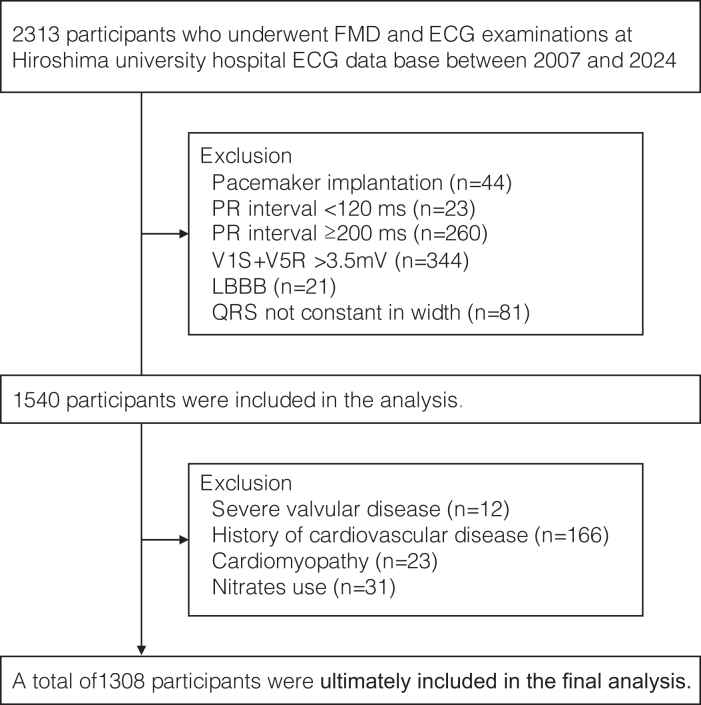
Flowchart of the Patient Selection Process A total of 2,313 individuals who visited the outpatient cardiology clinic or underwent health screening at Hiroshima University Hospital between August 2007 and March 2024 were screened. Among them, individuals who underwent vascular function tests and electrocardiography were evaluated. Participants were excluded based on predefined ECG abnormalities, structural heart diseases, prior cardiovascular interventions, inadequate ECG recordings, and nitrate use. Finally, 1,308 participants were included in this study. Of these, 1,253 individuals had a normal QRS duration, and 55 were identified as having complete right bundle branch block. ECG = electrocardiographic; FMD = flow-mediated vasodilation; LBBB = left bundle branch block.

This retrospective cross-sectional study included a total of 2,313 individuals who visited the outpatient cardiology clinic or underwent a health screening examination at Hiroshima University Hospital between August 2007 and March 2024. Vascular function tests and electrocardiography (ECG) were performed on the patients who provided informed consent for the vascular function examination. The exclusion criteria were as follows: patients with pacemaker implantation (n = 44); with PR interval of <120 milliseconds (n = 23); with PR interval of ≥200 milliseconds (n = 260); with V_1_S + V_5_R of >3.5 mV (n = 344); with QRS duration of ≥120 milliseconds with complete left bundle branch block pattern (n = 21); with inadequate electrocardiographic recording for the measurement of QRS duration (n = 81) due to atrial fibrillation (n = 72), atrial flutter (n = 8), and Wolff-Parkinson-White syndrome (n = 1); with severe valvular disease (n = 12); undergoing catheter-based cardiovascular treatment (n = 166); with cardiomyopathy (n = 23) including amyloid cardiomyopathy (n = 17), dilated cardiomyopathy (n = 3), and hypertrophic cardiomyopathy (n = 3); and treated with nitrates (n = 31). Finally, 1,308 individuals (561 men and 747 women; mean age: 56.8 ± 14.2 years) were enrolled in this study (Figure 1). Among them, 1,253 individuals had a normal QRS duration, and 55 individuals had CRBBB.

In the study, a normal QRS duration was defined as <120 milliseconds. CRBBB is diagnosed when the QRS duration is ≥120 milliseconds, and the QRS duration shows characteristic patterns such as rsr′, rsR′, or rSR′ in leads V_1_ or V_2_. In rare cases, a QR pattern may also be observed in these leads. The R′ deflection is typically wider than the initial R-wave. Additional findings include an S-wave of greater duration than the R-wave or >40 milliseconds in leads Ⅰ and V_6_ and a normal R-peak time in leads V_5_ and V_6_ but >50 milliseconds in lead V_1_. These criteria are based on the American College of Cardiology/American Heart Association Task Force on Clinical Practice Guidelines and the Heart Rhythm Society guidelines.^9^^,^^10^ Hypertension was defined as a treatment with oral antihypertensive drugs or systolic blood pressure of ≥140 mm Hg and/or diastolic blood pressure of ≥90 mm Hg measured in a sitting position on at least 3 different occasions without medication.^20^ Dyslipidemia was defined based on the third report of the National Cholesterol Education Program.^21^ Diabetes mellitus was defined based on the American Diabetes Association recommendation.^22^ Hyperuricemia was defined as treatment with oral uric acid–lowering drugs or a serum uric acid level of ≥7.0 mg/dL.^23^ Moreover, smoking was defined as current smoking. All methods were performed in accordance with the Declaration of Helsinki and relevant guidelines and regulations. The protocol was registered in the University Hospital Medical Information Network Clinical Trial Registry (UMIN000039512). Written informed consent for participation in the study was obtained from all individuals. The study protocol was approved by the ethics committee of Hiroshima University (registration number for clinical trial: E2022-0131)

### Electrocardiographic measurements

Standard 12-lead ECGs were obtained with patients in a supine position on beds with adequate surrounding space. The ECGs were recorded using digital devices (Nihon Kohden Co) at a paper speed of 25 mm/s and a sampling rate of 500 Hz. The raw digital data were stored using the Prime Vita data management system (Nihon Kohden Co).

### Measurements of FMD and NID

FMD and NID of the brachial artery were measured using UNEXEF18G (UNEX Co), an ultrasound instrument specialized for FMD and NID measurements, equipped with an automated edge detection system for measuring brachial artery diameter. The Supplemental Methods show the detailed information on the study protocol and measurement procedures for FMD and NID.

In brief, FMD was assessed by placing an occlusion cuff around the forearm and inflating to 50 mm Hg above systolic blood pressure for 5 minutes to induce reactive hyperemia, during which the brachial artery diameter was continuously measured. NID was assessed by measuring the change in brachial artery diameter in response to the sublingual administration of a 75-μg nitroglycerine tablet.^24^

All participants were instructed to fast for at least 8 hours and to abstain from caffeine, alcohol, smoking, and vigorous exercise before testing. The vascular measurements were performed in a quiet, temperature-controlled room in the morning, as in previous studies.^25^

These measurements were performed using the methods recommended in the guidelines for FMD and NID.^14^ Previous studies have shown that these standardized techniques generally provide acceptable inter- and intraobserver reproducibility.^26^ These measurements were performed by experienced cardiologists and technicians certified by the Japanese Society of Ultrasonics in Medicine, further confirming the reliability of the data. Investigators performing FMD and NID measurements were blinded to participants’ electrocardiographic findings and clinical data to minimize potential measurement bias.

### Statistical analysis

All reported *P* values were 2-sided, and *P* < 0.05 was considered statistically significant. The continuous variables were expressed as mean ± SD or median (Q1-Q3), depending on their distribution. Comparisons of variables with approximately normal distributions were performed using unpaired Student’s *t*-test. Categorical variables were expressed as frequencies and percentages and were compared using the chi-square test.

Correlations between continuous variables were assessed using the Pearson correlation coefficient, and 95% CIs for the correlation coefficients were estimated using Fisher *z*-transformation.

Endothelial dysfunction was defined as an FMD value below the cutoff between the lowest and middle tertiles (<3.3%). Vascular smooth muscle dysfunction was defined as an NID value below the cutoff between the lowest and middle tertiles (<10.9%).

A multivariable logistic regression analysis was performed to determine the independent variables associated with endothelial dysfunction and vascular smooth muscle dysfunction. The following variables were entered into the model: age ≥65 years, sex, body mass index, heart rate, serum sodium, serum potassium, serum chloride, hypertension, dyslipidemia, diabetes mellitus, hyperuricemia, and current smoking.

As a sensitivity analysis, propensity score matching was performed to minimize the selection bias in the evaluation of the relationship between CRBBB and vascular function. The variables included in the multivariable logistic regression model were prespecified based on clinical relevance and prior evidence, rather than selected using data-driven stepwise approaches. The propensity score was measured for each patient using logistic regression analysis based on the clinical variables, including age, sex, total cholesterol, low-density lipoprotein cholesterol, hypertension, and current smoking. Using these propensity scores and applying a caliper width of 0.25 SDs of the logit of the propensity score, 2 well-matched groups were created for the comparison of vascular function.

The quality of matching was assessed using standardized mean differences for all covariates, with an absolute standardized mean difference <0.1 indicating a good balance. Because propensity score matching was performed as a sensitivity analysis, matched data were analyzed using standard statistical methods rather than conditional or mixed-effects models.

A subgroup analysis was performed to assess endothelial function after excluding patients who were taking β-blockers or statins to evaluate the potential influence of these medications.

All data were processed using the JMP Pro version 16.0 software (SAS Institute).

## Results

### Baseline characteristics of the participants

Table 1 shows the baseline clinical characteristics of the 1,308 participants. The mean QRS duration was 92.0 ± 13.3 milliseconds. Of the 1,308 participants, 1,019 (77.9%) had hypertension, 919 (70.3%) had dyslipidemia, 374 (28.6%) had diabetes mellitus, and 255 (19.5%) had hyperuricemia, whereas 172 (13.1%) were current smoking. The median FMD and NID values were 4.5% (2.5%-6.7%) and 13.1% (9.5%-17.2%), respectively.

**Table 1:** Clinical Characteristics in Individuals With Normal QRS Duration and Individuals With CRBBB

	Total (N = 1,308)	Normal QRS Duration (n = 1,253)	CRBBB (n = 55)	OR (95% Cl)	*P* Value	SMD
Age, y	56.8 ± 14.2	56.6 ± 14.0	62.6 ± 16.6	1.03 (1.01-1.05)	0.002	0.391
Men	561 (42.9)	527 (42.1)	34 (61.8)	2.24 (1.27-3.97)	0.004	0.402
Body mass index, kg/m^2^	24.5 ± 4.6	24.5 ± 4.6	24.5 ± 5.2	1.00 (0.94-1.06)	0.997	0.000
Systolic blood pressure, mm Hg	130.1 ± 17.9	129.9 ± 17.8	134.1 ± 18.3	1.01 (1.00-1.03)	0.087	0.233
Diastolic blood pressure, mm Hg	79.2 ± 12.4	79.3 ± 12.5	77.1 ± 11.2	0.98 (0.96-1.01)	0.186	0.185
Total cholesterol, mg/dL	197.9 ± 41.0	199.2 ± 40.9	172.0 ± 33.9	0.98 (0.97-0.99)	<0.001	0.724
Triglycerides, mg/dL	151.3 ± 97.1	152.1 ± 96.2	134.1 ± 113.5	1.00 (0.99-1.00)	0.196	0.171
HDL cholesterol, mg/dL	59.0 ± 16.8	59.2 ± 17.0	55.4 ± 12.6	0.99 (0.97-1.00)	0.114	0.254
LDL cholesterol, mg/dL	116.5 ± 33.9	117.2 ± 33.6	102.5 ± 36.7	0.99 (0.98-0.99)	0.003	0.418
Serum sodium, mEq/L	140.2 ± 2.3	140.2 ± 2.3	140.3 ± 2.4	1.01 (0.90-1.14)	0.886	0.043
Serum potassium, mEq/L	4.1 ± 0.5	4.0 ± 0.5	4.1 ± 0.5	1.39 (0.77-2.51)	0.274	0.200
Serum chloride, mEq/L	104.9 ± 2.5	104.9 ± 2.5	104.8 ± 2.3	0.99 (0.89-1.11)	0.893	0.042
Glucose, mg/dL	111.6 ± 30.5	111.3 ± 29.7	117.7 ± 42.1	1.00 (1.00-1.01)	0.148	0.176
Hemoglobin A1c, %	5.6 ± 0.9	5.6 ± 0.9	5.7 ± 0.9	1.13 (0.82-1.44)	0.381	0.111
Uric acid, mg/dL	5.4 ± 1.4	5.4 ± 1.4	5.7 ± 1.5	1.14 (0.94-1.39)	0.178	0.207
Comorbidities						
Hypertension	1,019 (77.9)	967 (77.2)	52 (94.5)	4.86 (1.51-15.7)	0.004	0.512
Dyslipidemia	919 (70.3)	881 (70.3)	38 (69.1)	1.06 (0.59-1.88)	0.818	0.026
Diabetes mellitus	374 (28.6)	355 (28.3)	19 (34.5)	1.33 (0.75-2.36)	0.320	0.134
Hyperuricemia	255 (19.5)	240 (19.2)	14 (25.5)	1.29 (0.68-2.44)	0.432	0.152
Current smoking	172 (13.1)	168 (13.4)	4 (7.3)	1.97 (0.70-5.53)	0.188	0.201
Medication use						
Antihypertensive drugs	778 (59.5)	743 (59.3)	35 (63.6)	1.20 (0.69-2.10)	0.447	0.088
β-blockers	101 (7.7)	96 (7.7)	5 (9.1)	1.22 (0.48-3.14)	0.676	0.050
Lipid-lowering drugs	430 (32.9)	406 (32.4)	24 (43.6)	1.60 (0.93-2.76)	0.088	0.232
Antidiabetic drugs	226 (17.3)	214 (17.1)	12 (21.8)	13.8 (0.71-2.66)	0.338	0.119
Antihyperuricemic drugs	56 (4.3)	52 (4.2)	4 (7.3)	2.46 (0.68-7.00)	0.108	0.133
ECG measurements						
Heart rate, beats/min	69.7 ± 12.0	70.0 ± 11.9	64.8 ± 12.8	0.96 (0.93-0.98)	0.002	0.421
PR interval, milliseconds	159.7 ± 17.5	159.4 ± 17.5	166.5 ± 16.0	1.02 (1.01-1.04)	0.004	0.423
QRS duration, milliseconds	92.0 ± 13.3	90.3 ± 10.3	132.4 ± 9.0	N/A	<0.001	4.353
QTc interval, milliseconds	423.2 ± 24.2	421.9 ± 22.3	452.6 ± 41.5	1.04 (1.03-1.05)	<0.001	0.922
Brachial artery diameter, mm	3.9 ± 0.7	3.9 ± 0.7	4.1 ± 0.7	1.04 (1.00-1.08)	0.042	0.286
FMD, %	4.5 (2.5-6.7)	4.6 (2.6-6.7)	3.2 (1.6-4.7)	0.82 (0.73-0.91)	<0.001	
NID, %	13.1 (9.5-17.2)	13.1 (9.6-17.3)	11.5 (7.9-14.9)	0.94 (0.88-0.99)	0.024	

Values are mean ± SD, median (Q1-Q3), or n (%), unless otherwise indicated. ORs for continuous variables are shown per unit increase, resulting in smaller values than categorical variables.

CRBBB = complete right bundle branch block; ECG = electrocardiographic; HDL = high-density lipoprotein; LDL = low-density lipoprotein; N/A = not applicable; SMD = standardized mean difference.

### Associations between CRBBB, FMD, and NID

**Figure 2:**
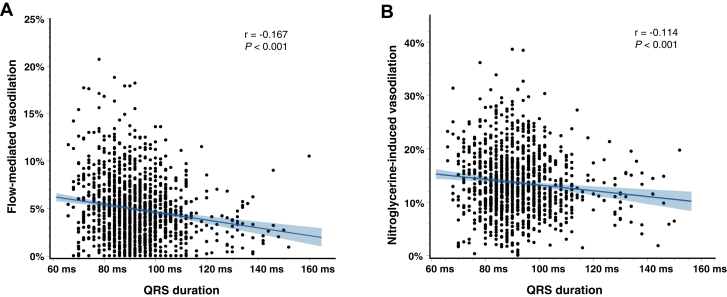
QRS Duration and Vascular Function Scatterplots show the correlations between QRS duration and flow-mediated vasodilation (FMD) (A) and nitroglycerine-induced vasodilation (NID) (B). Correlations were assessed using Pearson correlation coefficients. The QRS duration was significantly and inversely correlated with FMD (*r* = −0.167; *P* < 0.001) and NID (*r* = −0.114; *P* < 0.001), indicating that a longer QRS duration was associated with impaired endothelial function and vascular smooth muscle function.

The QRS duration was significantly correlated with FMD (*r* = −0.167; OR, 0.82; 95% CI, 0.73-0.91; *P* < 0.001) (Figure 2A) and NID (*r* = −0.114; OR, 0.94, 95% CI: 0.88-0.99; *P* < 0.001) (Figure 2B).

**Figure 3:**
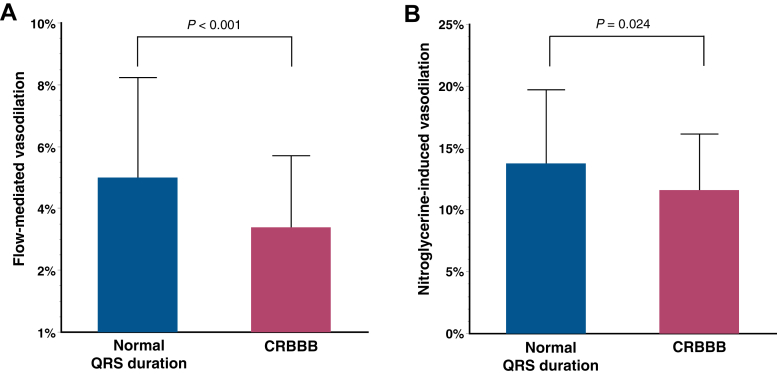
Vascular Function in CRBBB and Normal QRS Duration Bar graphs show FMD (A) and NID (B) in individuals with CRBBB and those with normal QRS duration. Values are presented as means, and error bars indicate SDs. Among the 1,308 participants, 55 had CRBBB. FMD was significantly lower in individuals with CRBBB than in those with normal QRS duration, indicating impaired endothelial function in individuals with CRBBB. NID was also significantly lower in the CRBBB group, suggesting concomitant impairment of vascular smooth muscle function in individuals with CRBBB. CRBBB = complete right bundle branch block. Other abbreviations as in Figure 2.

Of the 1,308 participants, 55 (4.2%) had CRBBB (Table 1). FMD was significantly lower in individuals with CRBBB than in those with normal QRS duration (3.2% [1.6%-4.7%] vs 4.6% [2.6%-6.7%]; *P* < 0.001) (Figure 3A). NID was significantly lower in individuals with CRBBB than in those with normal QRS duration (11.5% [7.9%-14.9%] vs 13.1% [9.6%-17.3%]; *P* = 0.024) (Figure 3B).

The multivariable logistic regression analysis revealed that CRBBB was significantly associated with endothelial dysfunction (OR: 1.83; 95% CI: 1.00-3.36; *P* = 0.049), whereas CRBBB was not significantly associated with vascular smooth muscle dysfunction (OR: 1.11; 95% CI: 0.52-2.28; *P* = 0.784) (Table 2).

**Table 2:** Multivariable Analysis of the Relationships Between CRBBB and Variables

	Endothelial Dysfunction		Vascular Smooth Muscle Dysfunction	
	OR (95% Cl)	*P* Value	OR (95% Cl)	*P* Value
Unadjusted	2.40 (1.38-4.08)	0.001	1.46 (0.78-2.76)	0.239
Model 1	1.91 (1.10-3.33)	0.022	1.20 (0.63-2.30)	0.577
Model 2	1.83 (1.00-3.36)	0.049	1.11 (0.52-2.28)	0.784

Model 1 is adjusted for age ≥65 y and sex. Model 2 is adjusted for age ≥65 y, sex, body mass index, heart rate, serum sodium, serum potassium, serum chloride, hypertension, dyslipidemia, diabetes mellitus, hyperuricemia, and current smoking. CRBBB = complete right bundle branch block; Endothelial dysfunction = division point for the lowest and under tertiles of flow-mediated vasodilation (<3.3%); Vascular smooth muscle dysfunction = division point for the lowest and tertiles nitroglycerine-induced vasodilation (<10.9%).

Propensity score matching was performed to create matched pairs between the 2 groups. Supplemental Table 1 shows the baseline clinical characteristics of the matched participants. FMD was significantly lower in patients with CRBBB than in individuals with normal QRS duration (3.2% [1.6%-4.1%] vs 4.1% [2.1%-6.6%]; *P* = 0.046) (Supplemental Figure 1A). However, no significant difference in NID was observed between the 2 groups (11.3% [7.1%-14.3%] vs 11.6% [8.3%-14.5%]; *P* = 0.498) (Supplemental Figure 1B).

After excluding patients who were taking β-blockers or statins, FMD was significantly lower in individuals with CRBBB than in those with normal QRS duration (3.3% [2.1%-5.5%] vs 4.9% [3.0%-7.1%]; *P* = 0.028) (Supplemental Figure 2B). NID was significantly lower in individuals with CRBBB than in those with normal QRS duration (11.5% [7.1%-14.7%] vs 13.9% [10.4%-18.0%]; *P* = 0.016) (Supplemental Figure 1B).

## Discussion

In this study, we demonstrated that patients with CRBBB exhibited significantly impaired vascular endothelial function, as assessed by FMD, compared with individuals with normal QRS duration. This association remained significant after adjusting for major cardiovascular risk factors, emphasizing a potential association between CRBBB and endothelial dysfunction, as summarized in the Central Illustration.

**Central Illustration:**
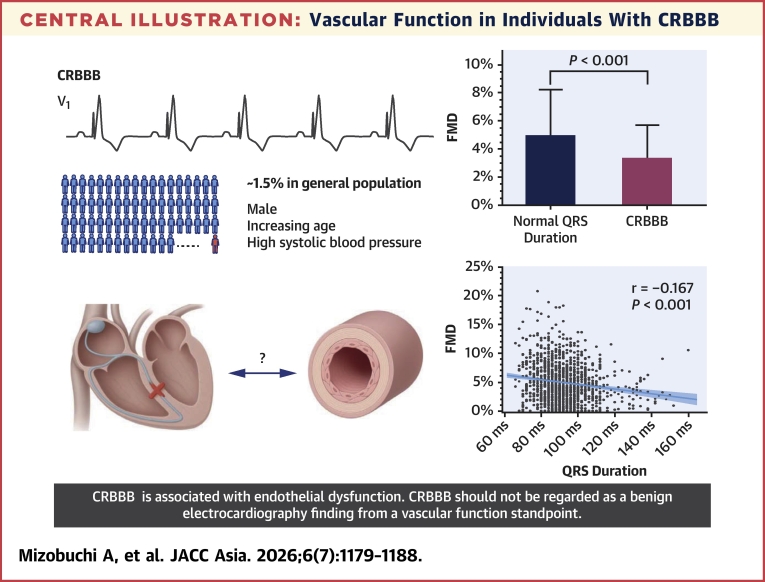
Vascular Function in Individuals With CRBBB The association between CRBBB and vascular function are summarized. Endothelial function was assessed using flow-mediated vasodilation (FMD), and vascular smooth muscle function was evaluated using nitroglycerine-induced vasodilation (NID). Individuals with CRBBB showed significantly impaired endothelial function compared with those with normal QRS duration, whereas there was no significant association between CRBBB and vascular smooth muscle dysfunction. These findings suggest that CRBBB should not be regarded as a benign electrocardiographic finding from the perspective of vascular function. CRBBB = complete right bundle branch block.

The prevalence of CRBBB has been reported to range from 0.1% to 8%, and CRBBB is considered a common electrocardiographic finding, particularly among men and older individuals.^2^^,^^27^, ^28^, ^29^ In the present study, CRBBB was observed in 55 participants (4.2%). The proportion of men and older individuals was significantly higher in individuals with CRBBB than in those with normal QRS duration.

The right bundle branch is an independent structure located on the distal side of the bundle of His. It is composed of Purkinje fiber cells, which have a fast conductive velocity.^30^ The right bundle branch spreads through the right ventricular (RV) endocardium of the ventricular septum, branching deep into the RV myocardium. The base of the right bundle branch is supplied by the left anterior descending coronary artery, whereas at the apex, the blood supply is provided by the right coronary artery and the left circumflex coronary artery, depending on the patient’s coronary artery vascularity. CRBBB is also known to be either congenital, due to abnormal development or anatomic variations of the conduction pathway, or acquired under certain pathologic conditions. Although the precise mechanisms underlying acquired CRBBB have not been determined, autopsy studies have demonstrated fibrosis of the right bundle branch tissue in patients with CRBBB.^31^

Myocardial infarction is one of the well-established causes of acquired CRBBB, reflecting both structural and ischemic damage to the conduction system. Moreover, CRBBB has been recognized as a marker of poor prognosis in the setting of acute myocardial infarction, often suggesting extensive myocardial injury and the involvement of the conduction system.^32^^,^^33^ This association is considered to be due to underlying anatomic factors. Newly developed CRBBB may result from the complete occlusion of the left anterior descending artery that supplies blood to the right bundle branch and the interventricular septum. Accordingly, the presence of CRBBB in the context of acute myocardial infarction may reflect extensive septal infarction.

In contrast, asymptomatic CRBBB is generally regarded as a benign electrocardiographic finding with a favorable long-term prognosis and, in most cases, does not require specific clinical management.^4^, ^5^, ^6^, ^7^, ^8^ However, in recent years, this perspective has been reevaluated in light of emerging evidence suggesting the potential associations of CRBBB with subclinical cardiac dysfunction and increased cardiovascular risk. Recent cohort studies have shown significant associations between CRBBB and the increased risks of all-cause and cardiovascular mortality.^1^^,^^4^^,^^28^ Alventosa-Zaidin et al^2^ reported that patients in whom incomplete right bundle branch block progressed to complete block showed a higher incidence of cardiovascular events.

Although the pathophysiological mechanisms underlying asymptomatic CRBBB have not yet been fully investigated, it is frequently observed in patients with hypertension.^1^^,^^8^^,^^34^ In the present study, hypertension was significantly more common in individuals with CRBBB than in those with normal QRS duration (52/55 [94.5%] vs 967/1,253 [77.2%]; *P* = 0.004). Our previous study reported a significant association between endothelial dysfunction assessed by FMD and hypertension.^35^ Several studies, including our own, have reported a significant association between endothelial dysfunction assessed by FMD and hypertension.^16^^,^^19^^,^^33^^,^^36^ FMD is also affected by age and sex, all of which overlap with the risk factors associated with CRBBB.

This study showed that the association between endothelial dysfunction and CRBBB remained significant after adjustment for potential confounders including age, sex, and hypertension. NID was also significantly lowered in individuals with CRBBB compared with those with normal QRS duration. However, the multivariable analysis revealed no significant association between CRBBB and vascular smooth muscle dysfunction. These findings suggest that CRBBB may serve as a significant risk factor for endothelial dysfunction. To our knowledge, this is the first study to report a significant association between CRBBB and endothelial dysfunction assessed using FMD.

Although the mechanisms underlying the association between CRBBB and endothelial dysfunction remain unclear, mechanical dyssynchrony caused by CRBBB may increase oxidative stress, which in turn could contribute to endothelial dysfunction. In support of this possibility, previous studies have reported that CRBBB-induced RV dyssynchrony may adversely affect RV size and function.^37^^,^^38^ Such changes in RV performance may reduce the pulmonary circulation efficiency and potentially influence systemic vascular regulation. CRBBB has also been shown to be associated not only with RV dyssynchrony and impaired contraction but also with reduced left ventricular global longitudinal strain and worsened mitral E/e' ratio.^38^ A decrease in global longitudinal strain has been reported to increase arterial load and contribute to endothelial dysfunction.^39^ These findings suggest that CRBBB might play a crucial role in endothelial dysfunction through a combination of mechanical and hemodynamic factors.

Although asymptomatic CRBBB has been traditionally regarded as a benign electrocardiographic finding, the results of this study suggest that its long-term prognosis and the need for follow-up or intervention should be reconsidered. A quantitative assessment of atherosclerosis in patients with CRBBB using FMD may serve as a useful option for patient follow-up. Our findings indicate that CRBBB is associated with impaired endothelial function, which is known to be associated with an increased risk of cardiovascular events. Therefore, patients with CRBBB may benefit from careful cardiovascular risk assessment and the proactive management of modifiable risk factors. Assessing endothelial function in these patients could serve as an additional tool to guide clinical decision-making and reduce cardiovascular risk.

### Study limitations

First, this study had a cross-sectional design and was conducted at a single institution, which may have limited the generalizability of the findings. Therefore, the results of our study need to be confirmed by a multicenter study of a large clinical trial. Although we assessed the relationship between CRBBB and endothelium-dependent vasodilatation assessed by FMD and endothelium-independent vasodilatation assessed by NID, we cannot determine a causal relationship.

Second, the number of participants with CRBBB was relatively small (n = 55), which may have compromised statistical power. Nonetheless, the overall sample size was sufficient, and the proportion of the CRBBB cases was comparable with previously reported prevalence (0.1%-8%), suggesting that the analyses still provide clinically meaningful insights.

Third, baseline brachial artery diameter and shear stress were not included in the statistical models. Because the primary aim of this study was to examine the association between CRBBB and endothelial function, we prioritized presenting FMD and NID to maintain clarity and interpretability. The inclusion of the baseline diameter or shear stress as covariates could have complicated the model and hindered interpretation.

Fourth, vascular function testing was performed only in participants who provided informed consent, which may have introduced selection bias.

Fifth, even though we adjusted for several major cardiovascular risk factors, the influence of medications cannot be entirely excluded. It is well known that β-blockers, statins, and antihypertensive agents influence vascular function. To further reduce the potential impact of these medications, a subgroup analysis was conducted excluding patients receiving β-blockers or statins. FMD and NID were still significantly lower in individuals with CRBBB than in those with normal QRS duration. Nevertheless, the potential effects of antihypertensive drugs cannot be completely excluded.

Finally, although CRBBB was associated with impaired endothelial function, the underlying mechanisms remain unclear. It is possible that unmeasured shared pathologic processes such as systemic fibrosis, inflammation, or oxidative stress may contribute to both conduction abnormalities and endothelial dysfunction. However, further research is needed to clarify these mechanisms.

## Conclusions

In this study, the findings suggest that CRBBB is associated with endothelial dysfunction and should not be regarded as a benign electrocardiographic finding from a vascular function standpoint.

## Funding Support and Author Disclosures

Dr Higashi has received support for this study by a grant-in-aid for scientific research from the Ministry of Education, Science and Culture of Japan (18590815 and 21590898). All other authors have reported that they have no relationships relevant to the contents of this paper to disclose.
