# Supplementary material for: Association Between Complete Right Bundle Branch Block and Vascular Endothelial Function
Source: JACC Asia. 2026 Apr 11;6(7):1179–88. doi: 10.1016/j.jacasi.2026.02.016 (PMC13350101; doi:10.1016/j.jacasi.2026.02.016)
Supplement: Supplemental Material [file mmc1.docx]

**Supplemental Material**

**Association Between Complete Right Bundle Branch Block and Vascular Endothelial Function**

Running title: CRBBB and endothelial function

Aya Mizobuchi, PhD;^1^ Tatsuya Maruhashi, MD, PhD;^1^ Yusuke Saito, MD;^1^ Takayuki Yamaji, MD, PhD;^1,2^ Takahiro Harada, MD, PhD;^1^ Farina Mohamad Yusoff, MBBS, PhD, FSVM;^1^ Shinji Kishimoto, MD, PhD;^1^ Masato Kajikawa, MD, PhD;^3^ Ayumu Nakashima, MD, PhD;^4^ Yukihito Higashi, MD, PhD, FAHA^1,2,3^

^1^Department of Regeneration and Medicine, Research Institute for Radiation Biology and Medicine, Hiroshima University, Hiroshima, Japan

^2^Center for Radiation Disaster Medical Science, Research Institute for Radiation Biology and Medicine, Hiroshima University, Hiroshima, Japan

^3^Division of Regeneration and Medicine, Medical Center for Translational and Clinical Research, Hiroshima University Hospital, Hiroshima, Japan

^4^Department of Nephrology, Graduate School of Medicine, University of Yamanashi, Yamanashi, Japan.

Address for correspondence: Yukihito Higashi, MD, PhD, FAHA

Department of Cardiovascular Regeneration and Medicine,

Research Institute for Radiation Biology and Medicine, Hiroshima University

1-2-3 Kasumi, Minami-ku, Hiroshima 734-8551, Japan

E-mail: [yhigashi@hiroshima-u.ac.jp](mailto:yhigashi@hiroshima-u.ac.jp)

**Supplemental Methods**

**Measurements of FMD and NID**

We measured the vascular response to reactive hyperemia in the brachial artery for assessment of endothelium-dependent FMD. A high-resolution linear artery transducer was coupled to computer-assisted analysis software (UNEXEF18G; UNEX Co., Nagoya, Japan) that used an automated edge detection system for measurement of the brachial artery diameter. A blood pressure cuff was placed around the forearm of each subject. The brachial artery was scanned longitudinally 5 cm to 10 cm above the elbow. When the clearest B-mode image of the anterior and posterior intimal interfaces between the lumen and vessel wall was obtained, the transducer was held at the same point throughout the scan by using a special probe holder (UNEX Co.) to ensure consistency of the imaging. Depth and gain setting were set to optimize the images of the arterial lumen wall interface. When the tracking gate was placed on the intima, the artery diameter was automatically tracked, and the waveform of diameter changes over the cardiac cycle was displayed in real time using the FMD mode of the tracking system. This allowed the ultrasound images to be optimized at the start of the scan and the transducer position to be adjusted immediately for optimal tracking performance throughout the scan. Pulsed Doppler flow was assessed at baseline and during peak hyperemic flow, which was confirmed to occur within 15 s after cuff deflation. The blood flow velocity was calculated from the color Doppler data and was displayed as a waveform in real time. Baseline longitudinal images of the artery were acquired for 30 s, and then the blood pressure cuff was inflated to 50 mmHg above systolic pressure for 5 min. The longitudinal image of the artery was recorded continuously until 5 min after cuff deflation. Pulsed Doppler velocity signals were obtained for 20 s at baseline and for 10 s immediately after cuff deflation. Changes in the brachial artery diameter were immediately expressed as the percentage change relative to the vessel diameter before cuff inflation. FMD was automatically calculated as the percentage change in peak vessel diameter from the baseline value. The percentage of FMD [(Peak diameter – Baseline diameter)/Baseline diameter] was used for analysis. Blood flow volume was calculated by multiplying the Doppler flow velocity (corrected for the angle) by heart rate and vessel cross-sectional area (-r ^2^). Reactive hyperemia was calculated as the maximum percentage increase in flow after cuff deflation compared with the baseline flow. The correlation coefficient between FMD analyzed at the core laboratory and participant institutions was 0.84 (P<0.001). The response to nitroglycerine was used for assessment of endothelium-independent vasodilation. After acquiring baseline rest images for 30 s, a sublingual tablet (nitroglycerine, 75 µg) was given and imaging of the artery was recorded continuously for 5 min. NID was automatically calculated as a percentage change in the peak vessel diameter from the baseline. The percentage of NID [ (Peak diameter - Baseline diameter) /Baseline diameter] was used for analysis. Inter- and intra-coefficients of variation for the brachial artery diameter were 1.6 and 1.4%, respectively, in our laboratory.

**Supplemental Table 1.** Clinical Characteristics of Propensity Score-matched Pairs of Individuals

|  | Normal  QRS duration | CRBBB | P value | SMD |
| --- | --- | --- | --- | --- |
| Variables | (n=45) | (n=45) |  |  |
| Age, yr | 63.4 ± 12.4 | 62.2 ± 16.5 | 0.707 | 0.082 |
| Men, n (%) | 18 (41.9) | 16 (37.2) | 0.659 | 0.096 |
| Body mass index, kg/m^2^ | 25.1 ± 4.9 | 25.2 ± 5.2 | 0.904 | 0.020 |
| Systolic blood pressure, mmHg | 131.6 ± 16.7 | 134.8 ± 18.1 | 0.403 | 0.184 |
| Diastolic blood pressure, mmHg | 78.5 ± 11.6 | 78.0 ± 11.5 | 0.838 | 0.043 |
| Total cholesterol, mg/dL | 178.7 ± 32.7 | 175.3 ± 32.5 | 0.633 | 0.104 |
| Triglycerides, mg/dL | 131.6 ± 74.3 | 138.4 ± 122.6 | 0.755 | 0.067 |
| HDL cholesterol, mg/dL | 57.4 ± 17.6 | 54.4 ± 12.0 | 0.346 | 0.199 |
| LDL cholesterol, mg/dL | 99.6 ± 27.7 | 101.6 ± 33.0 | 0.771 | 0.066 |
| Serum sodium, mEq/L | 140.1 ± 3.0 | 140.4 ± 2.2 | 0.577 | 0.114 |
| Serum potassium, mEq/L | 4.0 ± 0.4 | 4.0 ± 0.5 | 0.992 | 0.000 |
| Serum crawl, mEq/L | 104.5 ± 3.1 | 104.7 ± 2.3 | 0.771 | 0.073 |
| Glucose, mg/dL | 108.2 ± 19.0 | 118.8 ± 44.1 | 0.178 | 0.312 |
| Hemoglobin A1c, % | 5.6 ± 0.8 | 5.7 ± 0.9 | 0.627 | 0.117 |
| Urinc acid, mg/dL | 5.4 ± 1.4 | 5.6 ± 1.4 | 0.521 | 0.143 |
| Comorbidities, n (%) |  |  |  |  |
| Hypertension | 43 (100.0) | 40 (93.0) | 0.779 | 0.388 |
| Dyslipidemia | 32 (74.4) | 31 (72.1) | 0.808 | 0.052 |
| Diabetes mellitus | 11 (25.6) | 14 (32.6) | 0.476 | 0.493 |
| Hyperuricemia | 8 (18.6) | 11 (25.6) | 0.587 | 0.169 |
| Current smoking, n (%) | 6 (14.0) | 4 (9.3) | 0.501 | 0.147 |
| Medication use, n (%) |  |  |  |  |
| Anti-hypertensive drugs | 34 (79.1) | 28 (65.1) | 0.149 | 0.316 |
| β-blockers | 4 (9.3) | 3 (7.0) | 0.717 | 0.084 |
| Lipid lowering drugs | 21 (48.8) | 19 (44.2) | 0.666 | 0.092 |
| Anti-diabetic drugs | 8 (18.6) | 10 (23.3) | 0.557 | 0.116 |
| Anti-hyperuricemia drugs | 2 (4.7) | 3 (7.0) | 0.570 | 0.098 |

CRBBB indicates complete right bundle branch block; SMD, Standardized Mean Difference; HDL, high-density lipoprotein; LDL, low-density lipoprotein; ECG, electrocardiography; HR, heart rate; N/A, not applicable.

All results are presented as mean ±SD.

**Supplemental**

**Figure 1**


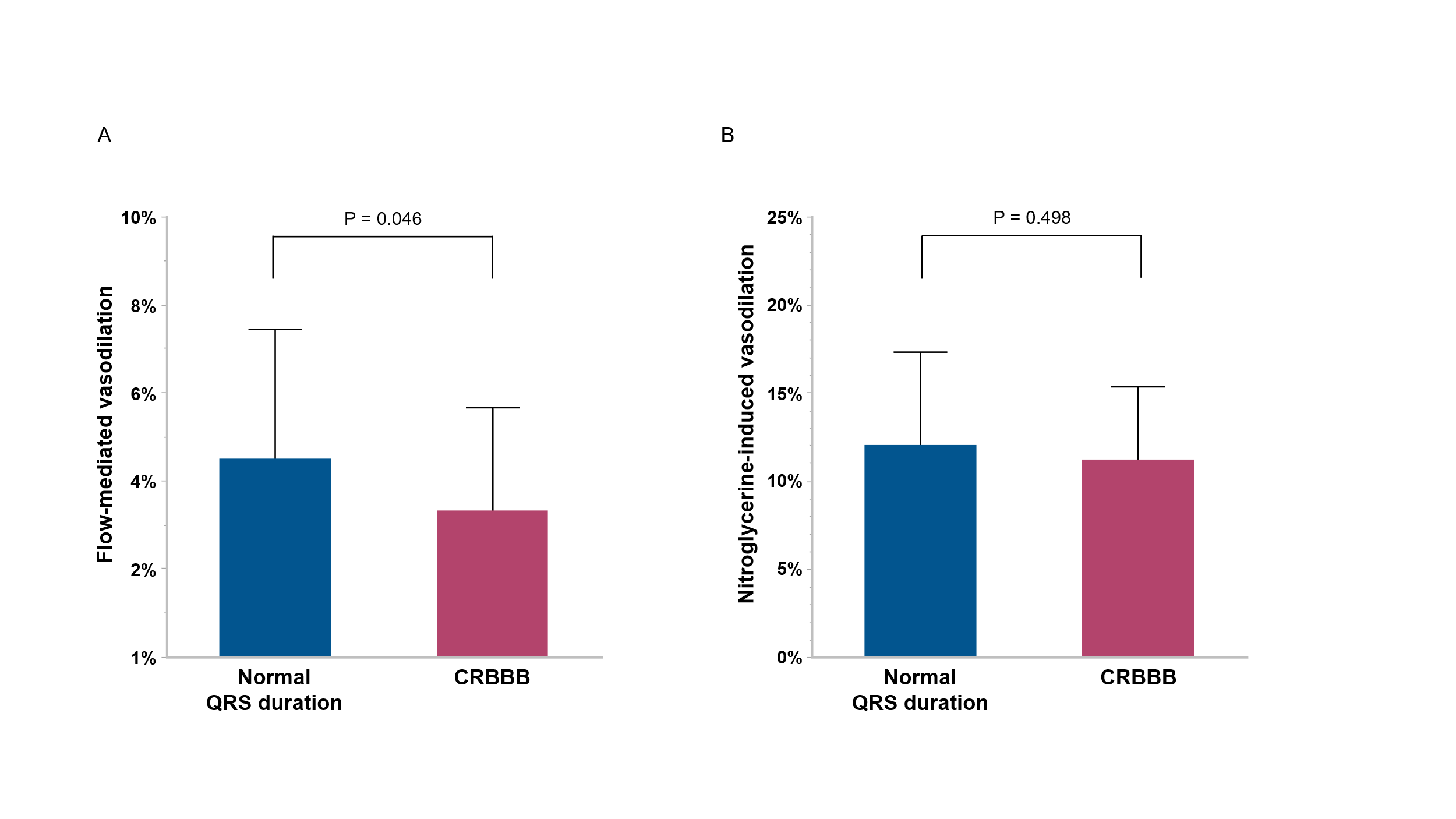


**Supplemental Figure 1.** Bar graphs show flow-mediated vasodilation (A) and nitroglycerine-induced vasodilation (B) of propensity score-matched pairs of individuals with complete right bundle branch block (CRBBB) and individuals with normal QRS duration.

**Supplemental Figure 2**


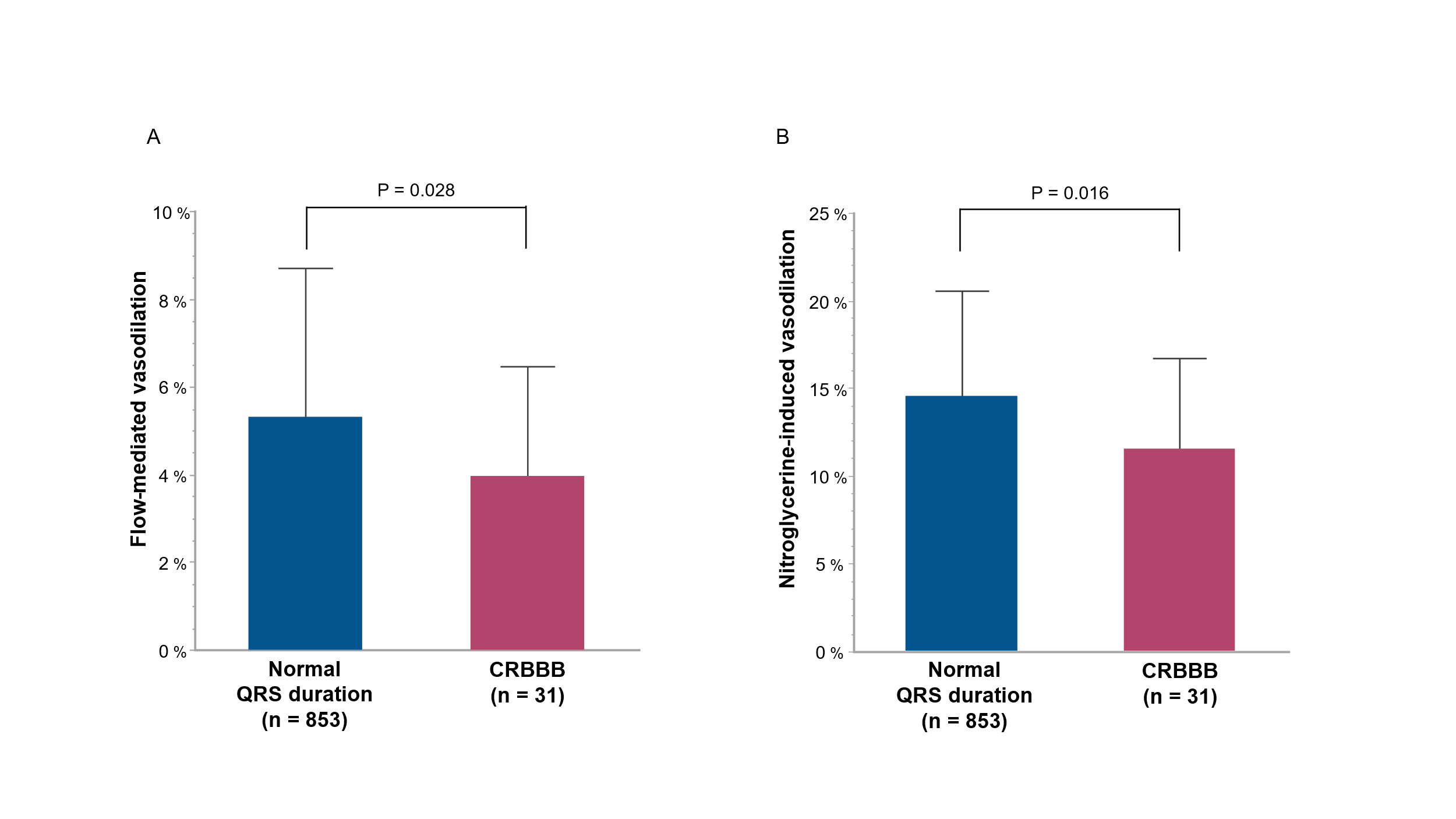


**Supplemental Figure 2.** Bar graphs show flow-mediated vasodilation (A) and nitroglycerine-induced vasodilation (B) in individuals with complete right bundle branch block (CRBBB) and individuals with normal QRS duration, after excluding patients taking statins or β-blockers.
